# Analysis of macular retinal thickness in polyarteritis nodosa using spectral domain optical coherence tomography

**DOI:** 10.1186/s12348-025-00453-1

**Published:** 2025-01-14

**Authors:** Che-Ning Yang, Chia-Ping Chen, Yi-Ting Hsieh

**Affiliations:** 1 School of Medicine, National Taiwan University, Taipei, Taiwan; 2 Department of Medical Education, National Taiwan University Hospital, Taipei, Taiwan; 3 Department of Ophthalmology, National Taiwan University Hospital, 7 Zhongshan S. Rd., Zhongzheng Dist., Taipei, 10002 Taiwan

**Keywords:** Polyarteritis nodosa, Optical coherence tomography, Retinal layer thickness, Retinal nerve fiber layer

## Abstract

**Purpose:**

To identify the macular retinal layer thickness changes in polyarteritis nodosa (PAN) patients without pathological findings appearing in color fundus photography (CFP), and to investigate the correlations with disease durations.

**Methods:**

A total of 24 PAN patients who had been for 3 years or more and underwent SD-OCT were recruited from the UK Biobank, with exclusions for diabetes, eye disease, or abnormal CFP findings. Only the right eyes were included, with each PAN patient paired one-to-one with a control matched for age, sex, and ethnicity. Paired t-tests or Wilcoxon Signed-Rank tests were used to assess the differences in thickness of different retinal layers between groups, followed by linear regression analysis to evaluate the correlations with disease durations.

**Results:**

PAN patients had significantly thinner retinal nerve fiber layer (RNFL) by 12.27% (mean ± standard deviation = 27.39 ± 8.94 μm for PAN patients and 31.22 ± 5.57 μm for controls, *p* = 0.048) and thinner outer plexiform and outer nuclear layers (OPL-ONL complex) by 10.67% (44.93 ± 6.59 μm for PAN patients and 50.31 ± 7.60 μm for controls, *p* = 0.032). Visual acuity and the whole macular thickness showed no statistical difference. The RNFL was thinned by 1.22 μm per year of disease progression (95% confidence interval: 0.12, 2.32, *p* = 0.042).

**Conclusions:**

PAN patients without visual impairments or abnormal CFP findings may exhibit significant thinning in RNFL and OPL-ONL complex. SD-OCT may serve as a useful tool for early screening of ophthalmic changes in PAN.

## Introduction

Polyarteritis nodosa (PAN) is a necrotizing vasculitis mostly affecting medium-sized arteries, sometimes small arteries, and usually antineutrophil cytoplasmic antibody (ANCA) negative [1, 2]. Endothelial dysfunction of medium vessels due to chronic inflammation may contribute to thrombosis and aneurysm formation, which are classic manifestation in PAN [3, 4]. Reported prevalence of PAN is 31 cases per million adults with male predominance [5]. Primary PAN is mostly idiopathic, while previous studies denoted association of hepatitis B virus infection and hairy cell leukemia in secondary PAN [6, 7].

As a systemic vasculitis, PAN may insult arteries in cardiovascular, gastrointestinal and nervous system, most commonly the renal arteries but sparing the lung [4]. Peripheral neuropathy and mononeuritis complex are the earliest symptoms and prevail at 50–70% in PAN patients [3, 8]. Eye involvement occurred in 10–20% of PAN patients, and ischemic retinopathy, branch and central retinal artery occlusion, anterior or posterior ischemic optic neuropathies, exudative retinal detachment, papilledema, papillitis, proptosis, episcleritis, scleritis, interstitial keratitis, and peripheral ulcerative keratitis have all been reported as ocular findings of PAN [9–13]. Retinal edema, hemorrhage and vasculitis can derive from the direct insult of retinal arterioles or indirect consequence of systemic hypertension [14–16]. Choroidal, conjunctival and optic nerve vascular supplies are also subjected to PAN involvement, with choroidal vasculitis being the most frequent ophthalmic findings in PAN patients [15, 17, 18]. Ischemia of retinal and choroidal arteries may result in retinopathy and neuropathy with additional findings of exudates and angiogenesis, which may eventually contribute to irreversible vision loss.

Optical coherence tomography (OCT), a in vivo, noninvasive tool for retinal imaging, has gained popularity not only in clinical practice but also among ophthalmologic researchers. OCT offers accurate measurement of retinal layers and microvasculature for evaluation. The quantitative data acquired by OCT greatly contribute to the early detection and diagnosis of neuro-ophthalmic diseases by measuring the thickness of each retinal layers and the presence of abnormal findings. The structural changes in OCT have been documented to precede the clinical manifestations in several retinal diseases. For example, hyperreflective foci, thinner inner nuclear layer (INL), thicker nerve fiber layer (RNFL) and interdigitation zone (IZ) could be detected by OCT in diabetic patients when no visible findings of diabetic retinopathy were noted under fundoscopy [19, 20]. Another example is hydroxychloroquine retinopathy, in which attenuation of the parafoveal ellipsoid zone and disruption of interdigitation zone continuity in OCT could be detected before the presence of clinically evident visual field defects or visual deterioration [21]. On the other hand, age, sex and ethnicity were reported to impact the retinal and choroidal thickness; interethnic differences were also revealed include the width of fovea, thickness of central foveal and inner retina [22].

To our best knowledge, there is no previous research exploring the neuroretinal morphological changes in PAN patients, although OCT can be used to detect subtle lesions before remarkable signs appear in color fundus photography (CFP). In this study, we aimed to identify the early structural alterations of the macula of PAN patients through macula-centered OCT imaging and the correlations with disease durations. We aspired to find signatural retinal layer thickness changes as indicators of PAN ophthalmological involvement.

## Methods

### Data collection

This study utilizes the data from the UK Biobank to explore the retinal structures of the PAN patients. The UK Biobank, established in 2006, is a large-scale biomedical database and research resources comprised of comprehensive health records and genetic information gathered from over 500,000 adults aged 40–69 across the UK [23]. It aims to facilitate the prevention, diagnosis and treatment of major diseases such as cancer, cardiovascular disease and more. Ophthalmological assessments such as visual acuity, intraocular pressure (IOP), refractive error, fundus photography, and OCT scans, were introduced in 2009 at selected medical centers. Macular-centered volume scans were performed in all patients using the Topcon 3D OCT-1000 Mark II (Topcon, Inc., Tokyo, Japan).

**Fig. 1:**
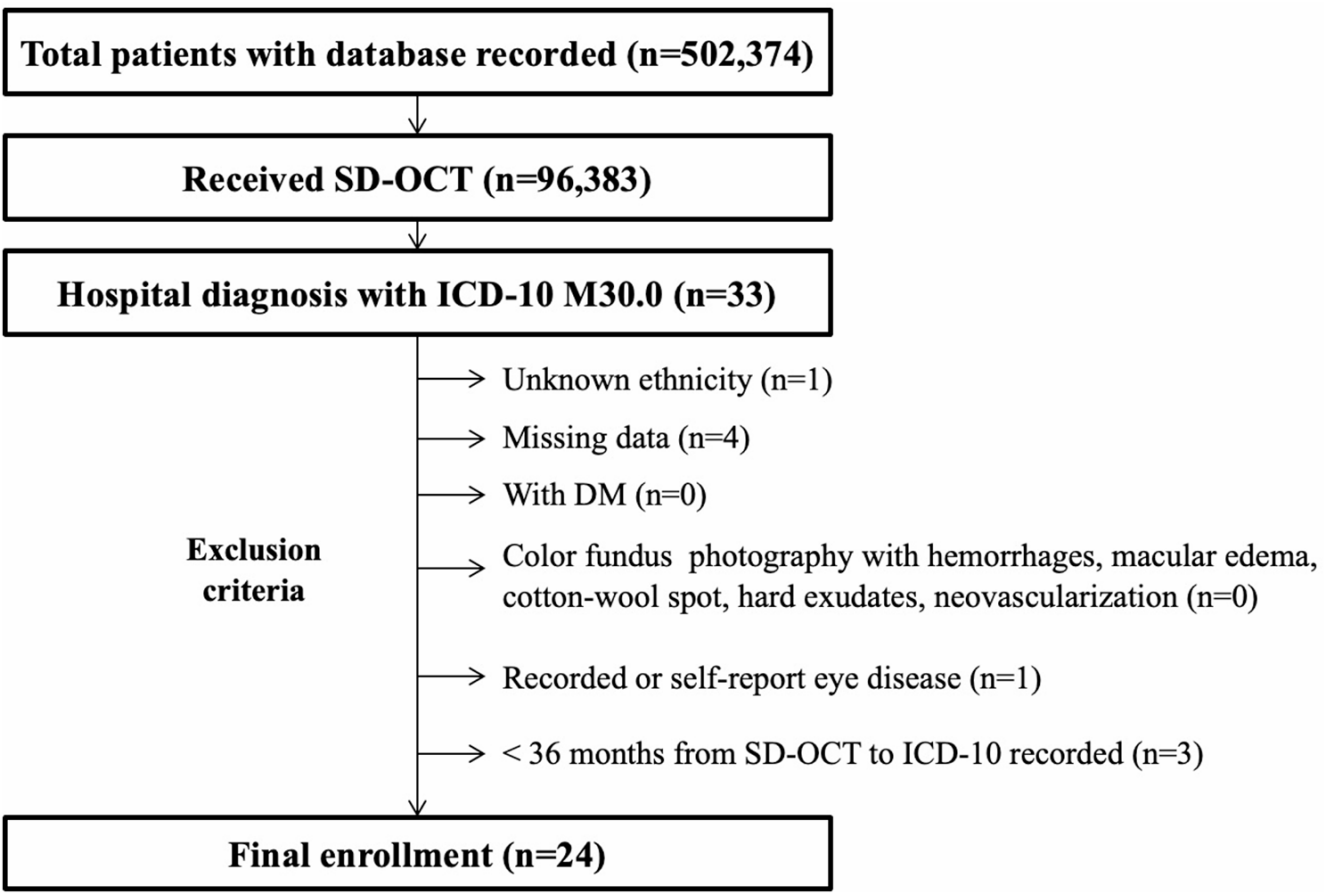
Flow chart for the inclusion of PAN patients from UK Biobank database. We screened the UK Biobank database for SD-OCT records, and then selected patients with ICD-10 diagnosis of PAN, then applied exclusion criteria to get the final enrollment subjects

We selected all the ICD-10 records of patients who had been diagnosed as PAN for more than 3 years prior to the time for OCT measurements. The duration of PAN was defined the term as the period between the receival of ICD-10 diagnosis of PAN and the date of OCT image acquisition. Exclusion criteria included patients with diabetes mellitus, recorded or self-report eye disease, patients with unknown ethnicity, missing data, and abnormal findings in CFP including hemorrhages, macular edema, cotton-wool spots, hard exudates or neovascularization, shown as Fig. 1. Only the right eyes of were included. For the control group, a same age, same sex, same ethnicity, healthy participant in the Biobank database who did not have diabetes mellitus, cardiovascular, neurological, systemic inflammatory diseases and ophthalmological diseases, was paired on a one-to-one basis. The study adhered to the tenets of the Declaration of Helsinki and was approved by the Institutional Review Board of National Taiwan University Hospital. (No.: 202304091 W).

### Measurements for retinal layer thickness

**Fig. 2:**
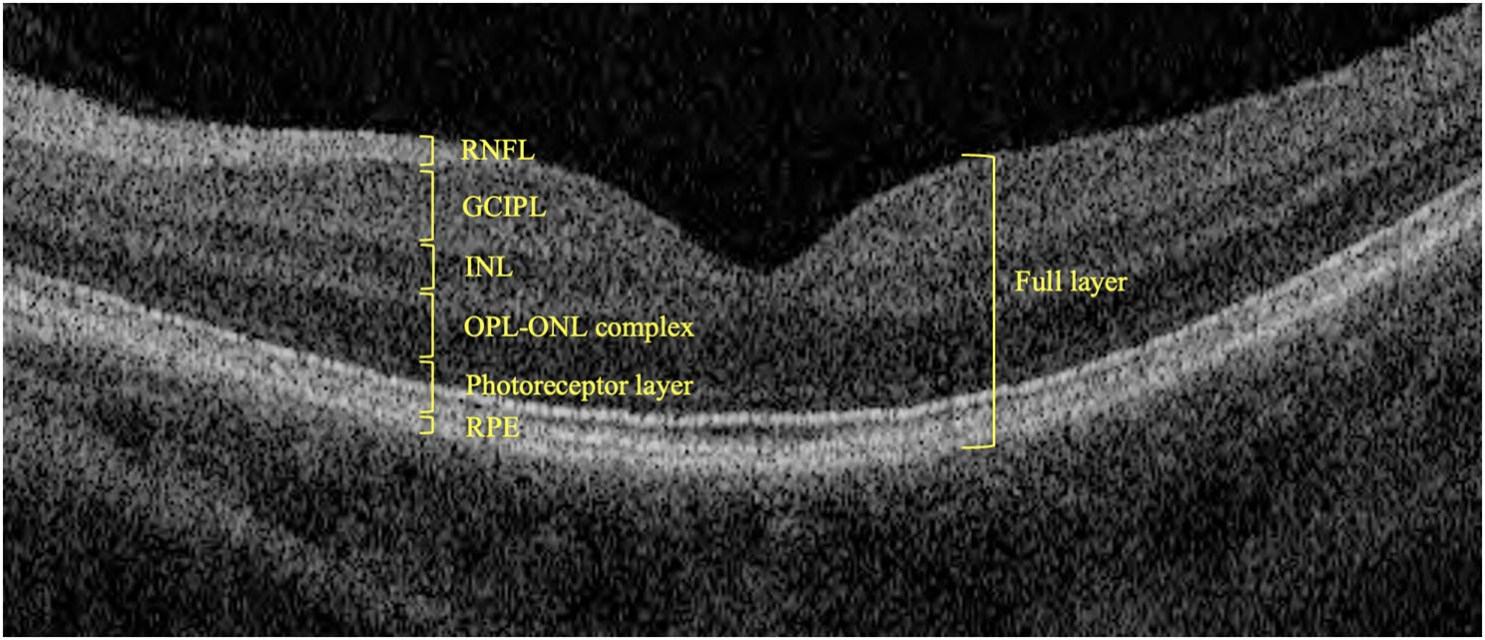
Diagram of the normal retina under optic coherence tomography with anatomical layer specified. Layers of retinal nerve fiber layer (RNFL), ganglion cell layer plus inner plexiform layer (GCIPL), inner nuclear layer (INL), outer plexiform layer and outer nuclear layer (OPL-ONL complex), photoreceptor layer, retinal pigment epithelium (RPE), and the full retinal layer were identified. All layers were measured the average thickness

For the retinal layer thickness, we collected the data that were measured automatically by the OCT machine, including the thickness of retinal nerve fiber layer (RNFL), ganglion cell layer plus inner plexiform layer (GCIPL), inner nuclear layer (INL), outer plexiform layer and outer nuclear layer (OPL-ONL complex), photoreceptor layer, retinal pigment epithelium (RPE), and the full retinal layer (Fig. 2).

### Statistical analysis

Mean and standard deviations (SD) of the visual acuity measured as logMAR, intraocular pressure (IOP), and thickness of different retinal layers were calculated. The Shapiro–Wilk test was employed to verify distribution normality for continuous variables. To determine statistically significant differences between groups, we used paired t-tests for normally distributed continuous variables, and the Wilcoxon Signed-Rank test was used for nonnormally distributed continuous variables. A two-tailed p-value less than 0.05 was considered statistically significant.

If a significant difference in the layer thickness is observed between the two groups, we performed linear regression on the difference, by subtracting the paired data, in relation to the disease duration to assess whether any correlation exists, with the duration as the independent variable. STATA, version 17 (Stata, College Station, TX, USA), was used for all statistical analyses.

## Results

### Demographic and clinical characteristics

A total of 24 patients diagnosed with PAN were included in this study, including 14 men and 10 women, with a mean age of 61.46 ± 5.20 years. The enrolled patients attended the assessment centers between 2010 and 2013, and all data were based on single OCT scans performed at the time of their attendance. Besides, all of them were of Caucasian ethnicity, while 23 were British and 1 was Irish. The mean duration of PAN among the patients was 7.63 ± 3.25 years. In comparison to the control group, the PAN patients did not exhibit statistically significant differences in age, sex, disease duration, visual acuity, or intraocular pressure (*p* > 0.05 for all) (Table 1). Furthermore, fundus images of all 24 PAN patients and healthy controls revealed no evidence of retinal vasculitis, vascular occlusion, macular edema, cotton-wool spots, or neo-vascularization.

**Table 1:** Demographic data, clinical characteristics and retinal layer thickness in patients with polyarteritis nodosa and normal controls

	PAN patients		Control group		Paired test
Variables	Mean ± standard deviation		Mean ± standard deviation		*p*-value
Age	61.46 ± 5.20		61.46 ± 5.20		1.00
Sex (male = 1)	58.33%		58.33%		1.00
Disease duration	7.63 ± 3.25		-		-
LogMAR	-0.05 ± 0.11		0.01 ± 0.15		0.29
IOP	16.62 ± 3.46		16.09 ± 3.70		0.76
Retinal layer thickness					
**RNFL**	27.39 ± 8.94		31.22 ± 5.57		**0.048** ^*^
**GCIPL**	68.20 ± 8.63		70.22 ± 8.79		0.32
**INL**	34.09 ± 5.34		32.69 ± 2.50		0.35
**OPL-ONL complex**	44.93 ± 6.59		50.31 ± 7.60		**0.032** ^*^
**Photoreceptor layer**	39.26 ± 6.97		35.17 ± 7.65		0.08
**RPE**	25.01 ± 2.92		25.12 ± 3.61		0.95
**Full layer**	272.98 ± 19.44		277.44 ± 14.59		0.63

*p < 0.05

RNFL: retinal nerve fiber layer, GCIPL: ganglion cell layer to inner plexiform layer, INL: inner nuclear layer, OPL-ONL complex: outer plexiform layer to external limiting membrane, RPE: retinal pigment epithelium, IOP: intraocular pressure

### Thickness in different retinal layers

**Fig. 3:**
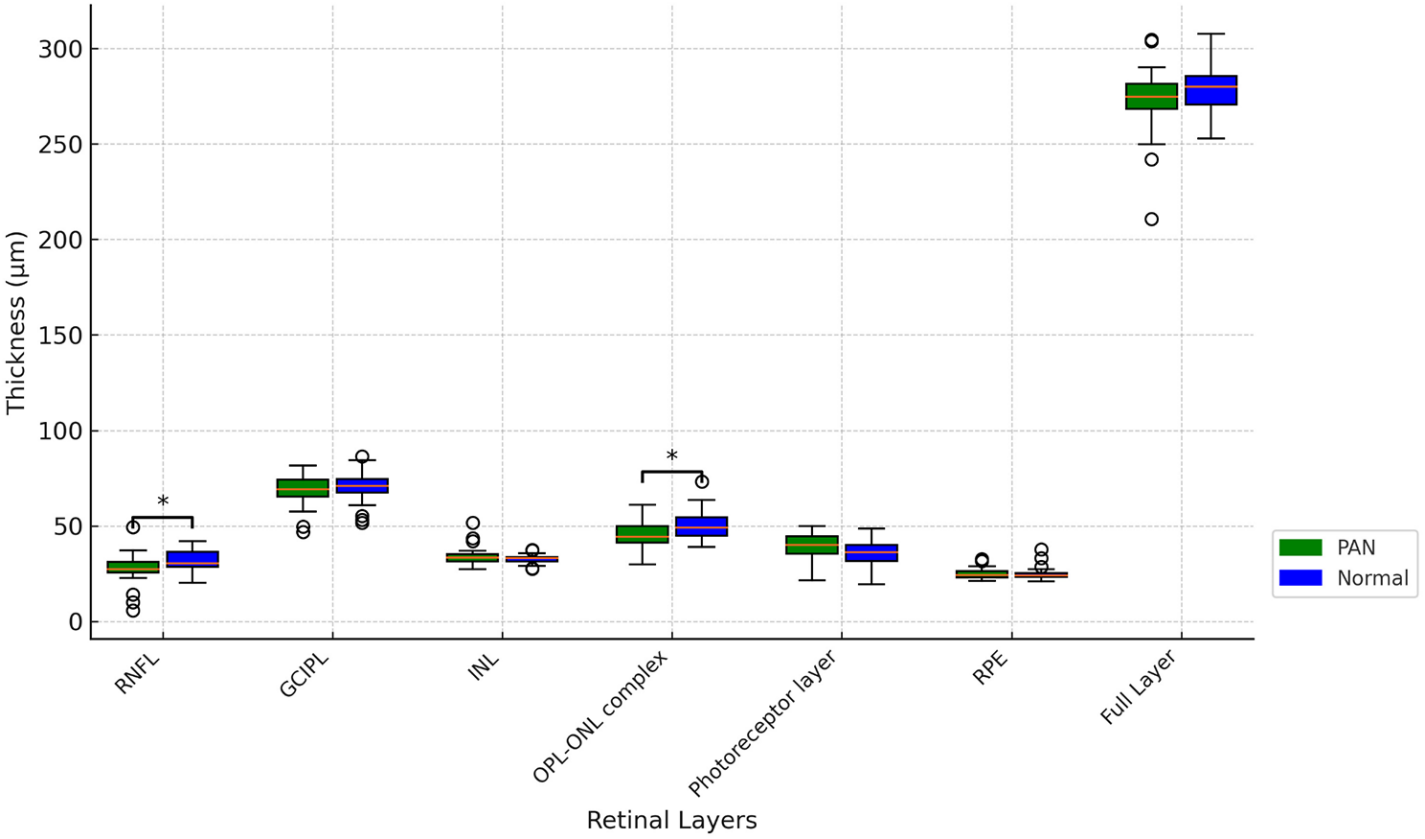
The box plot of measured retinal layer thickness of PAN patients and control group, where RNFL and OPL-ONL complex were revealed significantly thinner in PAN patients

The respective thicknesses of different retinal layers for both the PAN patients and the control group are presented in Table 1. The RNFL in PAN patients (27.39 ± 8.94 μm) was found to be 12.27% thinner than in the control group (31.22 ± 5.57 μm, *p* = 0.048). The OPL-ONL complex was 10.69% thinner in PAN patients compared to controls (44.93 ± 6.59 μm for PAN patients and 50.31 ± 7.60 μm for controls, *p* = 0.032). There were no significant differences in thickness in the ganglion cell-inner plexiform layer (GCIPL) (68.20 ± 8.63 μm for PAN patients and 70.22 ± 8.79 μm for controls, *p* = 0.32), inner nuclear layer (INL) (34.09 ± 5.34 μm for PAN patients and 32.69 ± 2.50 μm for controls, *p* = 0.35), photoreceptor layer (39.26 ± 6.97 μm for PAN patients and 35.17 ± 7.65 μm for controls, *p* = 0.08), RPE (25.01 ± 2.92 μm for PAN patients and 25.12 ± 3.61 μm for controls, *p* = 0.95), or full layer (272.98 ± 19.44 μm for PAN patients and 277.44 ± 19.50 μm for controls, *p* = 0.63). The box plots of measured retinal layer thickness of both PAN patients and control group were shown as Fig. 3.

The linear regression analysis revealed that RNFL thinning was significantly associated with a longer duration of PAN, with the *p*-value is 0.042. For each additional year of disease progression in PAN, the RNFL is expected to thin by 1.22 μm per year, with a 95% confidence interval of (0.12, 2.32). However, no significant correlation was found between the thickness of the OPL-ONL complex and PAN duration (*p* = 0.50).

## Discussion

In this study, thinning of the RNFL and OPL-ONL complex in PAN patients were observed using SD-OCT, based on the cohort of 24 patients aged 51 to 69 years. The RNFL and OPL-ONL complex were found to be 12.27% and 10.69% thinner, respectively, in PAN patients compared to the control group. Additionally, a significant association was identified between RNFL thinning and the duration of PAN, with a rate of 1.22 μm reduction in thickness per year. Upon searching previous studies, increased mean choroidal thickness and subfoveal choroidal thickness of PAN patients compared to healthy controls were observed, while the choroidal vascularity index showed no significant difference [24, 25]. Besides, the foveal avascular zone (FAZ) area and perimeter observed in OCT angiography were found to be smaller in PAN patients. These findings indicate that PAN patients may experience subtle structural changes in the choroid and retina, possibly reflecting the underlying inflammatory or ischemic processes. Though none of them investigated macular layers thickness in PAN patient, these retinal and choroidal may suggest the macular thickness changes found in our study not surprisingly. To our knowledge, this is the first study to analyze the changes of macular layers thickness in patients with polyarteritis nodosa.

Several previous studies have explored macular layers thickness in other systemic vasculitis conditions. In Bechet’s disease, recurrent vasculitis causes inflammatory and ischemic retinal damage. Cheng et al. found that inner retinal thickening during active inflammation, particularly in the ganglion cell layer (GCL) and inner nuclear layer (INL), potentially due to edema and vascular leakage [26]. In systemic lupus erythematosus (SLE), retinal thinning can occur even without ocular symptoms, indicating subclinical involvement [27]. However, the changes in RNFL thickness vary across studies, with some reporting significant thinning while others report non-significant findings [28, 29]. Besides, in giant cell arteritis (GCA), ischemic retinal damage from inflammation and posterior ciliary artery occlusion may cause anterior ischemic optic neuropathy. Some review and case report studies have revealed that thinning of the RNFL and GCL occurred, potentially due to axonal loss and ganglion cell death. Severe cases may also show thinning of the OPL and ONL from impaired choroidal perfusion [30, 31]. Compared to our results, while thinning of the RNFL and OPL-ONL complex was observed in PAN patients, changes in macular layer thickness were not entirely consistent, such as both PAN and GCA showed RNFL thinning, but GCL thinning occurred only in GCA. This indicates that the underlying pathological mechanisms contributing to these results may vary in these systemic vasculitis diseases. Furthermore, these studies also suggested that OCT is an essential tool for investigating ocular involvements of systemic vasculitis diseases and serves as effective indicators for monitoring disease progression.

As the RNFL is composed of retinal ganglion cell axons and functions as a signal transmitter from rod and cone cells, its thickness may, to some extent, reflect the overall health of the RNFL. Numerous previous studies have supported this association that RNFL thinning has also been observed in diabetic neuropathy [32], multiple sclerosis [33], systemic lupus erythematosus [29], hydroxychloroquine toxicity [21], Parkinson’s disease [34], Alzheimer’s disease [35], and hypertensive glaucoma [36]. Among them, Dehghani and colleagues revealed a reduction in RNFL thickness in patients with type 1 diabetes mellitus (T1DM) who presented with peripheral neuropathy, as measured by SD-OCT [37]. They observed a significant decline in overall RNFL thickness (− 0.7 μm per year, *p* = 0.02) in the T1DM group with diabetic peripheral neuropathy compared to those without. In contrast, retinopathy, diabetes duration, hemoglobin A1c levels, lipid profile, and blood pressure did not exhibit any significant effects on RNFL thickness. Peripheral neuropathy, commonly observed in patients with diabetes mellitus (DM), is also one of the earliest and most prevalent manifestations in individuals with PAN. The observed association between RNFL thickness and peripheral neuropathy may provide an explanation for our findings.

In addition, our subjects showed no significant differences in visual acuity compared to the control group, nor did they exhibit any pathological changes on color fundus photography. Although RNFL thinning is widely associated with poor visual acuity in many studies [38, 39], Fisher et al. demonstrated significant RNFL thinning in the eyes of multiple sclerosis (MS) patients, yet these patients still maintained excellent visual acuity, with median Snellen acuity equivalents better than 20/20. However, MS patients with thinner RNFL performed worse on low-contrast letter acuity and contrast sensitivity tests, supporting RNFL thickness as a structural biomarker of visual function [40]. The findings of Fisher’s study aligned closely with the results of this research, indicating that it is reasonable to expect normal visual acuity in the early stages of the disease, even with RNFL thinning. Thus, RNFL thinning may be inferred as an early indicator for assessing disease progression. Moreover, our result of a significant correlation observed between RNFL thinning and PAN duration, with a yearly reduction of 1.22 μm, further supports the potential of RNFL thinning as a reliable biomarker to monitor PAN progression.

Our results also revealed that the OPL-PNL complex was thinner in patients with polyarteritis nodosa compared to normal controls. A previous study by S. Reed et al. reported that MS patients without a history of optic neuritis presented significant thinning of GCL, IPL, OPL and ONL under OCT, while MS patients with a history of optic neuritis exhibited ONL thinning as time progressed. This inflammatory optic neuropathy is consistent with the clinical manifestations of PAN and may contribute to OPL and ONL thinning, in addition to ischemic stress [41]. Furthermore, patients with acute macular neuroretinopathy exhibited significantly thinner ONL, accompanied by perilesional thickening of the OPL [42]. In comparison to our findings, collective thinning of the OPL-ONL complex observed in PAN patient, it could be hypothesized that the transitional blood supply between the retinal and choroidal vasculature may increase the susceptibility of the OPL and ONL to ischemic events, leading to progressive thinning over time. This hypothesis was also supported by the study conducted by Lee et al., reporting significant thinning of the inner retina OPL and ONL in patients with central retinal vein occlusion [43]. Moreover, normal retinal circulation, along with delayed choroidal filling and staining of the affected arterial segments indicative of arteritis, has been emphasized in previous studies as a characteristic FA finding in PAN patients [44]. The delayed choroidal filling may further support the hypothesis, as both the OPL and ONL are primarily nourished by choroidal capillaries.

This study had several limitations. First of all, due to the rarity of PAN, the number of eligible patients was limited, which may have reduced the statistical power of our analysis and increased errors. Secondly, direct comparisons with previous studies were challenging due to the lack of respective OPL and ONL thickness data in the UK Biobank database. It would be even more analyzable whether subfields of RNFL were measured. Thirdly, the data was extracted from a retrospective dataset. A longitudinal follow-up of a specific cohort of patients over several years would provide deeper insights into the disease’s progression and underlying causes. Furthermore, information regarding the treatment status of PAN patients was not available. Immunosuppressive agents and systemic corticosteroids are commonly used therapeutic options [45], both of which can directly impact retinal changes [46, 47]. A comparison between patients who responded to treatment and those who did not would provide more meaningful insights. Last but not least, further robust research is needed to confirm our observational findings.

In conclusion, this essay highlights the potential of using OCT imaging to detect early ocular involvement in PAN patients, revealing thinning of the RNFL and OPL-ONL complex before structural or vascular changes are visible on CFP. To our knowledge, this is the first study to compare macular retinal layer thickness in PAN patients and healthy controls using quantitative OCT data. These findings emphasize the importance of routine ocular examinations by rheumatologists and ophthalmologists, allowing for early detection and intervention before PAN-related neuropathy or retinopathy progresses. With a single OCT scan, ophthalmologists can monitor retinal layer dynamics, providing a valuable tool for assessing disease progression in PAN patients.

## Data Availability

No datasets were generated or analysed during the current study.
